# Recovery of Central Fever after GH Therapy in a Patient with GH Deficiency Secondary to Posttraumatic Brain Injury

**DOI:** 10.4274/jcrpe.1639

**Published:** 2015-03-05

**Authors:** Hale Ünver Tuhan, Ahmet Anık, Gönül Çatlı, Ayhan Abacı, Tülay Öztürk, Handan Güleryüz, Ece Böber

**Affiliations:** 1 Dokuz Eylül University Faculty of Medicine, Department of Pediatric Endocrinology, İzmir, Turkey; 2 Dokuz Eylül University Faculty of Medicine, Department of Pediatric Radiology, İzmir, Turkey

**Keywords:** hypopituitarism, traumatic brain injury, central fever, Growth hormone deficiency, growth hormone therapy

## Abstract

Growth hormone (GH) deficiency is the most common pituitary hormone deficiency after traumatic brain injury. Patients with GH deficiency have a reduced sweating capacity which increases the risk of developing hyperthermia. To the best of our knowledge, central fever that developed due to GH deficiency and improved with GH treatment has not yet been reported. In this article, we present a case of dramatic improvement of central fever with recombinant human GH therapy in a patient with posttraumatic GH deficiency.

## INTRODUCTION

Hypopituitarism is a partial or complete insufficiency of pituitary hormone secretion that may result from pituitary or hypothalamic disorders. Various congenital and acquired pathologies may lead to hypopituitarism (1). Traumatic brain injury (TBI) is a rare cause of hypopituitarism (2). Growth hormone (GH) deficiency is the most common pituitary hormone deficiency in TBI patients (3,4).

Patients with GH deficiency have a reduced sweating capacity which increases the risk of developing hyperthermia (5). The differential diagnosis for fever of unknown origin includes autoimmune, neoplastic, metabolic, infectious, periodic, granulomatous and thermoregulatory disorders. It is believed that hypothalamus is the temperature regulatory centre of the brain. Damage to the thermoregulatory centers of the hypothalamus leads to central fever. To the best of our knowledge, development of central fever due to GH deficiency and its successful management with GH treatment has not been reported previously. In this article, we present a case of dramatic improvement of central fever with recombinant human GH (rhGH) therapy in a patient with posttraumatic GH deficiency.

## CASE REPORT

A seven-year-old boy who was involved in a traffic accident presented to our Emergency Unit in a state of coma as a result of head trauma.

The patient was born by spontaneous vaginal delivery after an uneventful pregnancy with a birth weight of 2750 g.

His growth and development were reported to be compatible with his chronological age.

**Figure 1:**
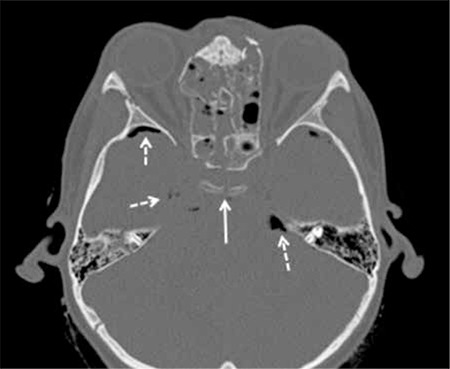
Axial unenhanced computed tomography image at the level of the orbits shows displaced fractures through the nasal bridge, ethmoid sinus walls, nondisplaced fracture through the right lateral orbital wall and depicts a fracture extension through the skull base and sella (arrow). Also, mix type fracture of the right temporal bone, loss of aeration of the right mastoid air cells and foci of pneumocephalus (dashed arrow) are observed

**Figure 2:**
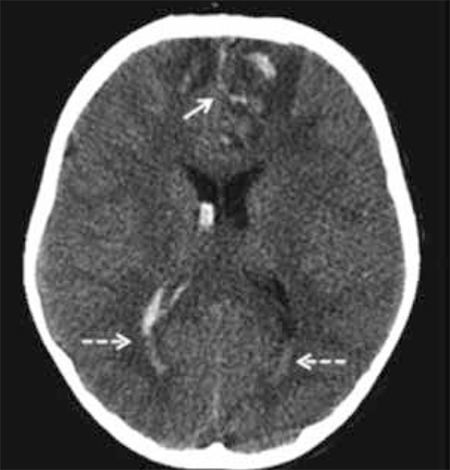
Axial nonenhanced computed tomography image shows hemorrhagic contusion areas in the frontal lobes bilaterally as foci of hyperdensity involving the grey matter and subcortical white matter and hyperdensities filling the subarachnoid space due to subarachnoid hemorrhage in the anterior interhemispheric fissure and sulci in the frontal lobes (arrow). In addition, intraventricular hemorrhage is seen (dashed arrow)

At the time of admission, the patient was unconscious (coma stage I, Glasgow scale), required intubation and mechanical ventilation. On physical examination, his weight was 25 kg [0.21 standard deviation score (SDS)] and his height was 118 cm (-1.20 SDS). Pubic hair was Tanner stage 1 and testicular size was 3 mL in both testes. Cranial computed tomography imaging showed multiple skull fractures, sella fracture, hydrocephalus, pneumocephalus and subarachnoid hemorrhage (Figures 1 and 2). On the second day of admission, the patient developed polyuria (2800 mL/m2/day) and hypernatremia (serum sodium 167 mmol/L with a paired urine density of 1004 g/cm3), thus the diagnosis of central diabetes insipidus (DI) was considered. Desmopressin treatment was started (2x10 ug/m2). Investigations for other pituitary hormone deficiencies revealed a state of central hypothyroidism [thyroid- stimulating hormone 0.31 µIU/mL (normal range: 0.35-5.6), free thyroxine 0.2 ng/dL (normal range: 0.61-1.12) and hypocortisolism: cortisol 2.3 µg/dL (normal range: 3.7-19.4)]. Hydrocortisone and L-thyroxine replacement treatment was initiated. He recovered consciousness two weeks after the TBI. The boy was taken to the service a month later and was followed for six months there.

**Figure 3:**
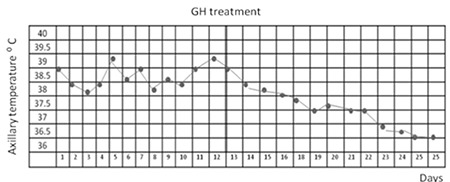
Fever chart of the patient

During the past six months in the hospital after the traffic accident, the patient had suffered from recurrent episodes of a prolonged febrile illness of unknown origin (Figure 3). His fever was ranging from 37.0 ˚C and 39.5 ˚C and was predominately at night without chills or sweating. All investigations related to infectious, autoimmune and neoplastic diseases were reported to be negative and the fever was considered to be of central origin.

On follow-up, the patient had a low height velocity [2 cm/year (-4.4 SDS)] and his serum insulin-like growth factor-1 (IGF-1) level was 50.3 ng/mL (-2.03 SDS). Serum IGF binding protein-3 (IGFBP-3) level was 1570 ng/mL (-3.45 SDS). Peak GH levels in GH stimulation tests with L-dopa and clonidine were 0.7 ng/mL and 0.6 ng/mL, respectively. A diagnosis of GH deficiency was considered and rhGH at a dose of 25 µg/kg/d was started. On the fifth day of the rhGH therapy, the patient’s body temperature returned to normal and he was still afebrile during the 3-month follow-up. 

## DISCUSSION

Although there is an increase in reports of hypopituitarism secondary to TBI, prevalence of endocrine dysfunction after TBI in children is not known. TBI-induced hypothalamic-pituitary damage may be due to direct injury to the hypothalamic-pituitary area or secondary injury from hypoxia or increased intracranial pressure (6). Our patient had a linear sellar fracture suggesting hypothalamic-pituitary damage. While TBI can affect all 7 pituitary hormones (7), GH deficiency is the most frequently reported pituitary hormone deficiency after TBI (3,4). Growth failure, delayed puberty, hyperphagia, temperature instability and polyuria are markers for possible pituitary dysfunction (8). These symptoms suggest GH, corticotropin, thyrotropin and antidiuretic hormone deficiencies which were all detected in our patient.

GH is an anabolic hormone that increases height in children (9). The hallmark symptoms of GH deficiency are decreased height velocity, muscle mass, bone density and increased central adiposity. Our patient had decreased height velocity, reduced IGF-1 and IGFBP-3 levels and an inadequate response to GH stimulation in two provocative tests.

The hypothalamic thermoregulatory center receives signals from peripheral and central thermal centers and radiates appropriate signals in the effector pathway. These signals affect the heat production, vasodilation and sweating and lead to loss of body heat through sweating (10). Several studies show that GH, either directly or through IGF-1, influences sweat secretion (5). GH receptors have been demonstrated in human eccrine sweat gland cells. Thus, GH may exert a direct effect on the eccrine sweat gland cells (11). Sweating is a known phenomenon of hyperpituitarism. It has recently been shown that patients with GH deficiency have a reduced sweating capacity which increases the risk of developing hyperthermia (5). Thus, the patients with GH deficiency might be at risk of developing hyperthermia secondary to disruption of sweat secretion (12). Also, it was reported that sweat secretion rate correlates significantly to serum IGF-1 levels in patients with GH deficiency (13). Our patient had recurrent episodes of a prolonged febrile illness of unknown origin during the six-month period preceding the admission to our clinic. All investigations related to infectious, autoimmune and neoplastic diseases were negative and the fever was characterized as central. Thus, his fever was considered to be a fever of central origin due to GH deficiency. GH deficiency was demonstrated by GH stimulation tests and rhGH treatment was started at a dose of 25 µg/kg/d. On the fifth day of rhGH treatment, the patient’s body temperature returned to normal and he remained afebrile during the follow-up.

Herein, we report a patient with a prolonged febrile illness of unknown origin, who presented with GH deficiency due to a previous head trauma. With this report, we would like to emphasize that in cases of TBI and fever of unknown origin, clinicians should keep GH deficiency in mind. These patients can be managed successfully with rhGH therapy.
